# Projections for lung cancer mortality in Brazil by 2045: a regional analysis

**DOI:** 10.1590/0102-311XEN199725

**Published:** 2026-07-24

**Authors:** Rejane de Souza Reis, Fernanda Cristina da Silva de Lima, Darlan Henrique Nascimento da Silva, Alfredo José Monteiro Scaff, André Salem Szklo

**Affiliations:** 1 Fundação do Câncer, Rio de Janeiro, Brasil.; 2 Instituto Nacional de Câncer, Rio de Janeiro, Brasil.

**Keywords:** Lung Neoplasms, Tobacco Use Disorders, Population Forecast, Mortality, Neoplasias Pulmonares, Tabaquismo, Prognóstico de Población, Mortalidad

## Abstract

The objective was to estimate temporal trends for lung cancer mortality in Brazil and its regions, stratified by sex and age group, from 2021 to 2045. This mortality projection study was based on lung cancer deaths (ICD-10: C33-C34) registered in the Brazilian Mortality Information System between 2001 and 2020, with redistribution of ill-defined causes. Crude and age-adjusted rates were calculated according to the 1960 world standard population. The projections adopted the *Nordpred* model in the R program, in 5-year periods from 2021 to 2045. There was a projected increase in the absolute number of deaths in both sexes, especially in the South and Southeast regions. The age-adjusted rates indicate a downward trend among men in all regions, despite remaining higher in the South. Among women, there was growth by 2025, followed by stability or a slight reduction by 2045, with the highest rates in the South Region. Population aging contributes to the increase in crude rates, even with the decline in adjusted rates. The projections suggest persistent regional inequalities, which may be associated with historical smoking patterns and unequal access to lung cancer diagnosis and treatment. The findings reinforce the need for regional prevention and control policies, including addressing smoking and considering the new forms of nicotine consumption, such as electronic cigarettes.

## Resumen

El objetivo fue estimar las tendencias temporales de la mortalidad por cáncer de pulmón en Brasil y sus regiones, estratificadas por sexo y rango de edad, desde el 2021 hasta el 2045. Se trata de un estudio de proyección de mortalidad basado en muertes por cáncer de pulmón (CIE-10: C33-C34) registrados en el Sistema de Información de Mortalidad entre el 2001 y el 2020, con redistribución de causas mal definidas. Se calcularon las tasas brutas y ajustadas por edad, con base en la población estándar mundial de 1960. Las proyecciones se realizaron utilizando el modelo *Nordpred* en el programa R, para períodos de cinco años comprendidos entre el 2021 y el 2045. Se proyectó un aumento en el número absoluto de muertes en ambos sexos, especialmente en las regiones del Sur y del Sudeste. Las tasas ajustadas por edad indican una tendencia a la baja entre los hombres en todas las regiones, aunque siguen siendo más elevadas en el Sur. Entre las mujeres, se observa un crecimiento hasta el 2025, seguido de estabilidad o un ligero descenso hasta el 2045, con las tasas más altas en la región Sur. El envejecimiento de la población contribuye al aumento de las tasas brutas, incluso con la disminución de las tasas ajustadas. Las proyecciones sugieren que persisten las desigualdades regionales, que pueden estar asociadas con los patrones históricos de tabaquismo y las diferencias en el acceso al diagnóstico y tratamiento del cáncer de pulmón. Los resultados refuerzan la necesidad de políticas regionales de prevención y control, que incluyan afrontar el tabaquismo y prestar atención a las nuevas formas de consumo de nicotina, como los cigarrillos electrónicos.

## Introduction

Cancer represents one of the leading causes of mortality worldwide, being responsible for approximately one in every six global deaths (16.8%) ^1^. Among the various types of cancer, lung cancer is notable as the leading cause of cancer death, with 1.8 million deaths recorded in 2022. It is estimated that, in the same year, there were about 2.5 million new cases of the disease worldwide ^2^.

Cancer incidence and mortality in general have increased globally, driven primarily by population aging ^3^ and exposure to modifiable risk factors, such as tobacco and alcohol use, inadequate diet, sedentary lifestyle, and air pollution ^4^ ^,^ ^5^.

In addition to its direct impact on health, cancer constitutes a significant social and economic challenge of the 21st century, representing a significant barrier to increased life expectancy. Its social and macroeconomic costs are significant and vary according to cancer type, geographic context, and gender disparities ^6^.

Brazil’s situation regarding lung cancer is concerning, with high rates for both incidence and mortality. According to estimates from the Brazilian National Cancer Institute, more than 32,000 new cases of the disease were expected for 2025 in the country ^7^. However, it is important to note that Brazil is a country of continental size, marked by major socioeconomic, cultural, and environmental diversity between its regions. These differences may reflect distinct profiles as to exposure to risk factors, access to health care services, and local cancer prevention and control strategies. Thus, a regional analysis is fundamental to better understand the epidemiological situation for lung cancer in the country, providing the identification of inequalities and inputs for more effective and equitable public policies.

Although there is already a projection study for lung cancer mortality in Brazil ^8^, there is still an important gap in the literature, with a lack of more recent and detailed analyses that consider the country’s regional differences. This fact reinforces the relevance and originality of the present study.

Estimates for new cancer cases and mortality are essential in supporting public policies and efficient distribution of resources geared toward addressing the disease. Cancer surveillance is fundamental for planning, monitoring, and assessing control strategies ^7^. Accordingly, the results of this study may serve as inputs for these specific policies, such as the Brazilian National Cancer Control Plan, supporting the identification of regional trends and more vulnerable population groups, providing better guidance for resource allocation, goal definition, as well as prioritization of initiatives for prevention, early detection, and timely treatment ^9^.

In Brazil, the last decade has been marked by advances in the quality and availability of information on cancer incidence and mortality. Within the scope of initiatives to address chronic non-communicable diseases, cancer surveillance - supported by information from population-based and hospital-based cancer registries, in addition to data from the Brazilian Mortality Information System (SIM, acronym in Portuguese) - enables public managers to organize and monitor interventions, in addition to orienting scientific production in the area ^10^.

The objective of this study was to estimate the projected temporal trends for lung cancer mortality rates, considering the country as a whole and its different geographic regions, stratified by sex and age group. We adopted the baseline period of 2001 to 2020 to analyze the trends for lung cancer mortality in Brazil. These trends supported projections for the period from 2021 to 2045, providing a comprehensive analysis of the expected evolution of the disease in the next decades.

## Materials and methods

This is a time-series ecological study, aiming to analyze trends and projections for lung cancer mortality in Brazil.

Mortality data were obtained from the SIM databases ^11^. The populations for Brazil and its regions were obtained using Brazilian Institute of Geography and Statistics (IBGE, acronym in Portuguese) databases ^12^. 

Mortality was analyzed according to the 10th revision of the International Classification of Diseases (ICD-10), for lung (C33-C34) and for ill-defined causes (R00-R99) ^13^. 

This study presents projections for lung cancer mortality in Brazil and its regions, disaggregated by sex and age group (0-39, 40-49, 50-59, 60-69, 70-79, and 80 years or older) for the years 2021 to 2045, grouped in 5-year periods ^14^. The projections were based on mortality historical series observed between 2001 and 2020, which constitute the model’s baseline period. The absolute number of deaths and the mortality rates - crude and age-adjusted by the 1960 world standard population - were estimated, providing a comprehensive view of the disease burden in the country ^14^ ^,^ ^15^. 

The denominator for calculating the mortality rates for Brazil and its regions was the census populations (2010), and, for the other years, the populations were from the IBGE population projection ^12^. 

The use of the Segi ^14^ standard population, follows the convention adopted in several national and international epidemiological studies, favoring temporal comparability with historical series and between countries that use the same standardization ^16^ ^,^ ^17^. Although more recent alternatives, such as the World Health Organization (WHO) standard population 2000-2025, may better reflect the contemporary age structure, their adoption could hinder comparisons with previous studies, especially those that analyzed long historical series for mortality. Crude rates directly reflect the absolute number of cases in relation to the total population, being influenced by population aging. Age-adjusted rates control for the effect of age structure, providing more appropriate comparisons over time and between regions. 

The model requires at least fifteen consecutive years of information (five-year periods). The projections were calculated for each period (2021-2025; 2026-2030; 2031-2035; 2036-2040; 2041-2045) using the *Nordpred* program, within the R statistical program (http://www.r-project.org) ^18^.

This function was developed by the Cancer Registry of Norway and is based on a version of the age-period-cohort (APC) model, widely used in projecting cancer incidence and mortality trends. The APC models were fitted considering the link function (power-5) and Poisson to assess the fit and possible overdispersion effects. The comparison was based on the deviance, degrees of freedom, and p-value of model ^18^ ^,^ ^19^ ^,^ ^20^. The results (Supplementary Material; https://cadernos.ensp.fiocruz.br/static//arquivo/supl-e00199725_4669.pdf) showed that both fits presented goodness of fit, with deviance/degrees of freedom ratios close to 1, indicating the absence of relevant overdispersion. The differences between the two link functions were small, and the power-5 model was adopted for the final projections. This transformation smooths the relationship between effects and rates, reducing the influence of extreme values and improving the stability of projections, especially in long series and with small annual fluctuations.

The model can be represented by the equation: Rap = (Aa + Dp + Pp + Cc)^5^, in which *Rap* is the mortality rate in age group *a* and period *p*; *D* is the common trend parameter (drift); *Aa*, the age component; *Pp*, the nonlinear period component; and *Cc*, the nonlinear cohort component ^19^ ^,^ ^20^.

The redistribution of ill-defined causes of death (categories included in Chapter XVIII of ICD-10: R00-R99) followed the methodology proposed by Mathers et al. ^21^, which assumes that the distribution of true causes of these deaths is the same as that of deaths reported for natural (non-external) causes. This redistribution was proportional among natural causes (excluding external causes - Chapter XX: V01-Y98) according to sex, five-year age group, and geographic region. For each year and state, correction factors were calculated and applied to well-defined causes, obtained based on the ratio between the total estimated deaths and the total reported deaths in the SIM ^21^ ^,^ ^22^. It was applied only to the observed period, with the objective of adjusting the historical series before modeling and projection. Therefore, the projections produced by the *Nordpred* model considered deaths already corrected for under-registration and ill-defined causes.

As per *Resolution n. 510/2016* of the Brazilian National Health Council, studies with secondary, publicly accessible data, are exempt from the need for ethical assessment.

## Results

The results show that both the absolute number and the crude mortality rates for lung cancer are expected to continue increasing until 2045, in both sexes, driven mainly by population aging and the growing burden of the disease in older age groups. Among men, there was an increase in the number of deaths from lung cancer over the analyzed periods. Deaths increased from 63,949 in 2001-2005 to 78,491 in 2016-2020 (Table 1). Projections indicate that this growth should continue, reaching 133,700 deaths in 2041-2045 (Table 2). Among women, the growth pattern was also observed. The number of deaths increased from 30,535 in 2001-2005 to 51,690 in 2016-2020, reaching 121,114 deaths in 2041-45 (Tables 3 and 4). 

**Table 1:** Observed number of deaths and crude rates for lung cancer mortality per 100,000 men. Brazil and regions, 2001 to 2020.

Regions/Age goups (years)	Observed number of deaths				Observed crude rates			
	2001-2005	2006-2010	2011-2015	2016-2020	2001-2005	2006-2010	2011-2015	2016-2020
North								
0-39	83	102	110	100	0.29	0.34	0.35	0.31
40-49	193	175	220	188	5.70	4.39	4.77	3.50
50-59	493	547	591	669	23.58	20.99	18.62	17.91
60-69	829	913	1,053	1,280	67.44	62.16	58.01	56.16
70-79	761	881	1,132	1,283	122.36	118.93	125.30	116.15
80 or more	320	417	622	726	120.70	137.84	172.94	163.26
Total	2,679	3,035	3,728	4,246	7.47	7.71	8.81	9.49
Northeast								
0-39	320	293	341	336	0.35	0.31	0.37	0.37
40-49	748	715	691	650	6.01	4.98	4.38	3.74
50-59	1,765	1,972	2,267	2,345	20.72	20.14	19.77	17.64
60-69	2,731	3,158	3,878	4,688	49.48	49.72	52.50	54.74
70-79	2,721	3,036	3,967	4,880	85.93	86.21	96.59	101.45
80 or more	1,434	1,831	2,393	3,154	90.40	103.48	124.27	144.18
Total	9,719	11,005	13,537	16,053	7.86	8.52	10.18	11.78
Central-West								
0-39	76	76	97	72	0.32	0.31	0.38	0.28
40-49	269	279	249	218	7.27	6.44	5.09	3.98
50-59	648	744	898	871	27.75	25.83	25.74	21.22
60-69	1,081	1,262	1,528	1,706	78.00	76.64	75.63	67.87
70-79	952	1,186	1,527	1,760	144.09	142.86	149.73	142.43
80 or more	415	537	748	1,014	167.02	174.90	195.26	205.94
Total	3,441	4,084	5,047	5,641	10.75	11.75	13.59	14.24
Southeast								
0-39	483	425	465	462	0.37	0.32	0.35	0.36
40-49	2,232	2,015	1,535	1,253	9.16	7.63	5.55	4.26
50-59	5,762	6,510	6,683	5,824	35.62	33.43	29.87	23.86
60-69	10,076	10,045	10,908	12,691	104.32	89.60	79.51	76.22
70-79	9,656	10,445	10,926	11,850	183.97	174.00	159.20	144.87
80 or more	3,643	4,756	5,977	7,508	201.35	208.80	213.75	221.35
Total	31,852	34,196	36,494	39,588	16.92	17.33	17.84	18.77
South								
0-39	179	188	183	176	0.40	0.42	0.42	0.40
40-49	1,053	1,022	734	575	12.28	10.90	7.60	5.77
50-59	3,057	3,486	3,543	3,168	53.03	50.51	44.29	35.85
60-69	5,504	5,804	6,219	6,924	161.24	143.68	125.19	114.68
70-79	4,852	5,621	6,086	6,548	274.38	273.60	252.34	220.60
80 or more	1,613	2,302	2,920	3,468	272.11	315.05	326.43	313.69
Total	16,258	18,423	19,685	20,859	25.13	27.30	28.11	28.56
Brazil								
0-39	1,141	1,084	1,196	1,146	0.36	0.33	0.37	0.36
40-49	4,495	4,206	3,429	2,884	8.57	7.20	5.48	4.27
50-59	11,725	13,259	13,982	12,877	33.61	31.83	28.83	23.68
60-69	20,221	21,182	23,586	27,289	95.35	85.69	78.86	75.71
70-79	18,942	21,169	23,638	26,321	165.20	160.98	154.44	143.84
80 or more	7,425	9,843	12,660	15,870	164.93	182.70	199.08	208.21
Total	63,949	70,743	78,491	86,387	14.39	15.12	16.12	17.12

**Table 2:** Projected number of deaths and crude rates for lung cancer mortality per 100,000 men. Brazil and regions, 2021 to 2045.

Regions/Age goups (years)	Projected number of deaths					Projected crude rates				
	2021-2025	2026-2030	2031-2035	2036-2040	2041-2045	2021-2025	2026-2030	2031-2035	2036-2040	2041-2045
North										
0-39	100	99	97	95	90	0.32	0.32	0.33	0.34	0.34
40-49	213	223	220	229	236	3.42	3.28	3.15	3.17	3.14
50-59	672	710	786	852	861	15.61	14.10	13.36	13.17	12.94
60-69	1,382	1,492	1,624	1,790	2,033	50.12	45.50	42.27	39.47	38.12
70-79	1,440	1,759	2,018	2,266	2,534	107.02	102.20	93.8	87.31	82.31
80 or more	863	1,009	1,240	1,578	1,877	164.88	152.65	144.3	139.84	129.78
Total	4,670	5,292	5,985	6,810	7,631	10.03	10.99	12.11	13.54	15.04
Northeast										
0-39	342	351	354	347	334	0.40	0.43	0.46	0.48	0.50
40-49	761	753	762	758	774	3.89	3.59	3.69	3.74	3.82
50-59	2,283	2,399	2,605	2,588	2,584	15.58	14.79	14.12	13.03	13.14
60-69	5,303	5,620	5,527	5,829	6,338	52.81	47.88	42.19	39.80	37.73
70-79	5,880	7,064	8,093	8,590	8,622	105.41	107.48	102.30	91.61	81.41
80 or more	3,710	4,638	5,808	7,109	8,339	150.87	155.34	159.65	160.34	152.19
Total	18,279	20,825	23,149	25,221	26,991	13.21	14.91	16.49	17.95	19.29
Central-West										
0-39	68	50	47	47	44	0.26	0.20	0.19	0.19	0.18
40-49	213	229	209	159	158	3.46	3.47	3.12	2.34	2.29
50-59	771	709	750	843	798	16.62	13.56	12.68	13.24	12.30
60-69	1,842	1,915	1,838	1,838	2,030	60.63	53.10	44.41	39.03	37.80
70-79	1,996	2,302	2,648	2,891	2,912	131.93	120.56	111.63	101.15	87.59
80 or more	1,135	1,372	1,699	2,075	2,498	191.28	183.07	175.08	164.74	155.12
Total	6,025	6,577	7,191	7,853	8,440	14.44	15.08	15.88	16.84	17.70
Southeast										
0-39	478	483	459	435	405	0.39	0.41	0.41	0.41	0.41
40-49	1,290	1,469	1,543	1,631	1,594	4.03	4.42	4.78	5.18	5.14
50-59	5,071	4,673	4,913	5,605	5,880	19.81	17.05	16.33	17.84	19.16
60-69	13,281	12,450	11,495	11,191	12,204	69.50	59.08	51.13	45.95	45.18
70-79	13,308	15,766	17,177	16,831	16,198	132.91	127.91	118.02	103.07	91.49
80 or more	7,811	8,900	10,773	13,345	15,210	199.21	181.32	170.45	166.21	154.92
Total	41,239	43,741	46,360	49,038	51,491	19.26	20.25	21.35	22.59	23.86
South										
0-39	160	156	145	137	126	0.37	0.37	0.35	0.35	0.33
40-49	633	669	659	697	677	5.86	5.78	5.59	5.87	5.77
50-59	2,518	2,263	2,543	2,801	2,862	27.48	23.76	24.47	25.10	25.12
60-69	7,149	6,743	5,862	5,716	6,557	101.75	86.05	71.19	66.16	68.96
70-79	7,186	8,217	9,030	8,946	8,151	194.34	179.66	165.27	144.68	123.83
80 or more	3,804	4,422	5,314	6,481	7,509	284.61	253.52	231.35	220.03	205.66
Total	21,450	22,470	23,553	24,778	25,882	28.37	28.89	29.67	30.82	32.00
Brazil										
0-39	1,148	1,139	1,102	1,061	999	0.37	0.38	0.39	0.39	0.39
40-49	3,110	3,343	3,393	3,474	3,439	4.16	4.22	4.33	4.47	4.44
50-59	11,315	10,754	11,597	12,689	12,985	19.39	16.96	16.4	16.86	17.34
60-69	28,957	28,220	26,346	26,364	29,162	68.99	59.37	50.87	46.35	45.55
70-79	29,810	35,108	38,966	39,524	38,417	134.6	129.54	120.07	105.84	93.06
80 or more	17,323	20,341	24,834	30,588	35,433	196.11	184.10	176.32	171.88	161.02
Total	91,663	98,905	106,238	113,700	120,435	17.75	18.83	19.98	21.26	22.51

**Table 3:** Observed number of deaths and crude rates for lung cancer mortality per 100,000 women. Brazil and regions, 2001 to 2020.

Regions/Age goups (years)	Observed number of deaths				Observed crude rates			
	2001-2005	2006-2010	2011-2015	2016-2020	2001-2005	2006-2010	2011-2015	2016-2020
North								
0-39	82	84	84	87	0.29	0.28	0.27	0.28
40-49	164	167	157	171	5.06	4.29	3.45	3.19
50-59	267	305	426	500	13.76	12.25	13.73	13.49
60-69	385	436	580	820	33.37	31.22	32.85	36.00
70-79	337	421	606	813	54.44	55.48	64.60	70.29
80 or more	197	262	371	499	60.02	70.53	83.43	90.53
Total	1,432	1,675	2,224	2,890	4.06	4.32	5.33	6.54
Northeast								
0-39	258	235	274	286	0.28	0.25	0.29	0.32
40-49	554	661	755	783	4.07	4.19	4.35	4.13
50-59	1,130	1,457	2,018	2,309	11.92	13.21	15.57	15.30
60-69	1,518	2,024	2,938	3,911	23.80	27.70	34.14	38.61
70-79	1,335	1,840	2,709	3,702	34.62	41.44	52.13	61.17
80 or more	849	1,200	1,833	2,730	40.89	49.22	65.07	82.22
Total	5,644	7,417	10,527	13,721	4.37	5.48	7.52	9.54
Central-West								
0-39	61	68	81	65	0.26	0.27	0.32	0.26
40-49	149	216	254	204	3.92	4.77	4.96	3.54
50-59	347	458	634	759	14.99	15.39	17.10	17.20
60-69	493	648	890	1,142	36.26	38.60	41.40	41.18
70-79	463	639	861	1,175	66.23	71.52	76.56	83.04
80 or more	258	358	560	709	83.80	93.53	114.84	110.89
Total	1,771	2,387	3,280	4,054	8.10	9.24	11.11	12.12
Southeast								
0-39	374	418	406	386	0.28	0.31	0.31	0.30
40-49	1,522	1,709	1,461	1,272	5.79	5.94	4.86	4.01
50-59	2,852	4,004	5,264	5,151	16.14	18.51	20.87	18.64
60-69	3,857	4,980	6,557	9,078	34.14	37.88	40.52	45.65
70-79	3,939	4,984	6,043	7,487	54.69	60.93	65.40	68.71
80 or more	2,356	3,338	4,403	5,807	75.49	83.04	88.90	98.20
Total	14,900	19,433	24,134	29,181	7.50	9.30	11.13	13.04
South								
0-39	138	157	161	136	0.31	0.36	0.37	0.31
40-49	618	729	763	598	6.88	7.34	7.46	5.71
50-59	1,343	1,769	2,315	2,640	22.05	23.90	26.61	27.26
60-69	1,929	2,598	3,229	4,204	50.39	57.26	57.45	60.90
70-79	1,849	2,398	3,205	3,901	78.74	88.53	102.53	103.02
80 or more	911	1,298	1,852	2,432	92.16	103.22	118.98	128.99
Total	6,788	8,949	11,525	13,911	10.17	12.80	15.83	18.29
Brazil								
0-39	913	962	1,006	960	0.28	0.29	0.31	0.30
40-49	3,007	3,482	3,390	3,028	5.38	5.54	5.04	4.19
50-59	5,939	7,993	10,657	11,359	15.84	17.56	19.85	18.77
60-69	8,182	10,686	14,194	19,155	34.07	38.07	41.35	45.64
70-79	7,923	10,282	13,424	17,078	53.81	60.55	68.40	73.28
80 or more	4,571	6,456	9,019	12,177	67.00	76.23	87.92	98.92
Total	30,535	39,861	51,690	63,757	6.61	8.16	10.15	12.07

**Table 4:** Projected number of deaths and crude rates for lung cancer mortality per 100,000 women. Brazil and regions, 2021 to 2045.

Regions/Age goups (years)	Projected number of deaths				Projected crude rates			
	2021-2025	2026-2030	2031-2035	2036-2040	2041-2045	2021-2025	2026-2030	2031-2035	2036-2040	2041-2045
North										
0-39	87	78	83	83	81	0.28	0.26	0.29	0.31	0.32
40-49	197	226	212	184	193	3.15	3.29	3.00	2.53	2.59
50-59	534	545	621	687	645	12.37	10.70	10.36	10.41	9.49
60-69	1,045	1,247	1,328	1,372	1,560	37.00	36.65	33.11	28.8	27.69
70-79	1,087	1,447	1,859	2,191	2,352	74.55	76.34	77.36	74.84	67.52
80 or more	675	928	1,261	1,683	2,215	102.02	111.39	115.02	115.09	116.00
Total	3,625	4,471	5,364	6,200	7,046	7.86	9.37	10.91	12.36	13.87
Northeast										
0-39	250	216	228	224	219	0.29	0.27	0.30	0.32	0.34
40-49	861	891	730	574	582	4.05	3.91	3.25	2.64	2.75
50-59	2,490	2,584	2,716	2,670	2,197	14.97	14.17	13.21	12.08	10.06
60-69	4,889	5,666	5,835	5,871	6,138	40.90	40.39	37.42	34.13	31.46
70-79	4,988	6,395	7,826	8,769	9,005	69.73	75.17	76.51	72.27	66.20
80 or more	3,637	4,947	6,559	8,326	10,293	95.63	108.25	116.99	121.25	121.16
Total	17,115	20,699	23,894	26,434	28,434	11.68	13.96	15.99	17.63	19.02
Central-West										
0-39	43	31	28	28	26	0.17	0.12	0.11	0.12	0.11
40-49	215	196	153	110	109	3.31	2.84	2.22	1.59	1.58
50-59	760	743	775	752	629	15.19	13.15	12.14	11.07	9.25
60-69	1,399	1,693	1,764	1,818	1,949	40.57	41.02	37.39	33.93	32.04
70-79	1,470	1,859	2,391	2,939	3,132	80.78	78.58	79.90	81.33	75.22
80 or more	934	1,281	1,729	2,290	3,034	115.57	120.24	120.86	120.09	122.24
Total	4,821	5,803	6,840	7,937	8,879	12.83	13.98	15.23	16.62	17.81
Southeast										
0-39	358	330	325	310	294	0.29	0.29	0.30	0.30	0.31
40-49	1,292	1,311	1,176	1,074	1,056	3.76	3.72	3.48	3.29	3.34
50-59	4,598	4,244	4,341	4,376	3,974	15.93	13.89	13.06	12.78	12.07
60-69	11,036	11,219	10,174	9,615	9,975	47.70	44.01	37.82	33.51	31.77
70-79	9,783	13,249	16,194	16,548	15,350	72.88	79.70	82.26	75.45	65.65
80 or more	6,921	8,749	11,617	15,898	20,127	102.69	106.92	111.83	120.46	124.45
Total	33,988	39,102	43,827	47,821	50,776	14.91	16.96	18.87	20.56	21.93
South										
0-39	145	153	148	141	132	0.34	0.37	0.37	0.37	0.36
40-49	616	634	631	682	668	5.46	5.30	5.23	5.64	5.67
50-59	2,426	2,199	2,249	2,349	2,369	24.18	21.3	20.14	19.84	19.83
60-69	5,210	5,744	5,501	5,167	5,390	64.02	63.01	57.82	52.47	50.39
70-79	4,803	6,221	7,853	8,732	8,494	101.4	105.99	111.75	109.95	101.72
80 or more	3,081	3,997	5,127	6,839	8,964	138.08	141.43	139.9	146.22	154.53
Total	16,281	18,948	21,509	23,910	26,017	20.62	23.28	25.83	28.3	30.59
Brazil										
0-39	883	808	812	786	752	0.29	0.28	0.29	0.3	0.31
40-49	3,181	3,258	2,902	2,624	2,608	3.99	3.89	3.53	3.25	3.30
50-59	10,808	10,315	10,702	10,834	9,814	16.67	14.77	13.84	13.28	12.22
60-69	23,579	25,569	24,602	23,843	25,012	47.63	45.52	40.51	36.2	34.11
70-79	22,131	29,171	36,123	39,179	38,333	77.41	82.73	85.32	80.7	72.35
80 or more	15,248	19,902	26,293	35,036	44,633	107.05	113.87	118.51	124.64	128.03
Total	75,830	89,023	101,434	112,302	121,152	13.98	16.10	18.08	19.86	21.39

When comparing the crude rates in Brazil from the last observed five-year period (2016-2020) with the last projected five-year period (2021-2045), an important increase is observed in both sexes. Among men, the rate increases from 17.12 to 22.51 per 100,000 men, representing an increase of approximately 31.5% in the period. Among women, the projected growth is even more significant: the rate increases from 12.07 to 21.39 per 100,000 women, corresponding to an increase of about 77.2% (Tables 1, 2, 3 and 4).

When analyzed regionally, it is observed that the South and Southeast regions presented the highest crude rates in all periods, reaching 32.00 and 23.86 deaths per 100,000 men in the final five-year period, respectively. The North maintains the lowest rates, with 10.99 to 15.04 deaths per 100,000 men between 2026 and 2045 (Table 2).

Although the crude mortality rate among women is lower in relation to men, there is also growth over time in Brazil. The South and Southeast regions once again are notable with the highest rates, reaching 30.59 and 21.93 deaths per 100,000 women, respectively, between 2041 and 2045. While the North region presented the lowest rates, with values between 9.37 and 13.87 deaths per 100,000 women in the period from 2026 to 2045 (Table 4).

Regarding age group-specific rates, distinct patterns are observed in Brazil according to age and sex. Among men, there is a decline in the younger age groups: for example, in the 40-49 age group, the crude mortality rate falls from 8.57 in 2001-2005 to 4.44 deaths per 100,000 men in 2041-2045 (Tables 1 and 2). Among women, similar behavior is observed in some age groups; between 40-49 years, it went from 5.38 to 3.30 deaths per 100,000 women, and in the 50-59 years age group, the rate decreases from 15.84 to 12.22 deaths per 100,000 women between 2001-2005 and 2041-2045 (Tables 3 and 4). In contrast, in the older age groups (over 70 years), the rates tend to remain high, while the number of deaths at these ages grows expressively. This set of results reinforces the inverse relationship between the reduction in the risk of death within various age groups and the increase in the total volume of deaths over the analyzed period.

**Figure 1:**
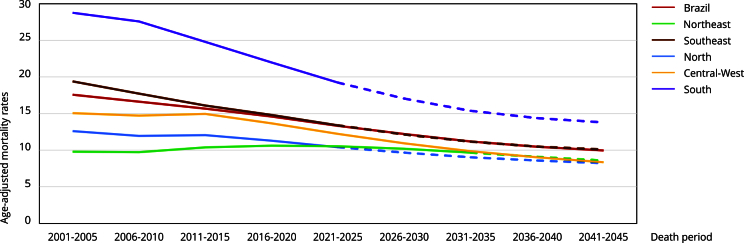
Age-adjusted * a mortality rates for lung cancer, per 100,000 men. Brazil and regions, 2001 to 2045.

Source: Brazilian Health Informatics Department ^11^ and Brazilian Institute of Geography and Statistics ^12^. Note: solid lines represent observed rates, dashed lines represent projected rates. * World standard population, 1960.

A downward trend in age-adjusted lung cancer mortality rates among men in Brazil is observed across all regions between 2001 and 2045. The South Region consistently shows the highest rates throughout the entire period, despite the projected progressive reduction. The North and Northeast regions maintain the lowest indices, with a more moderate decline (Figure 1).

**Figure 2:**
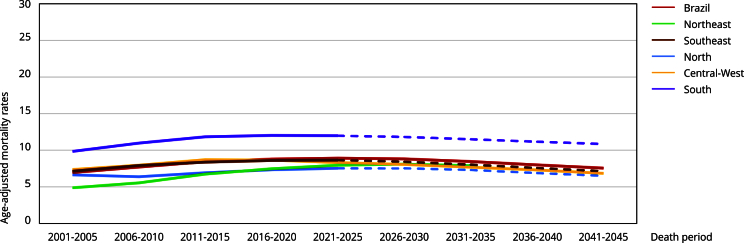
Age-adjusted * mortality rates for lung cancer, per 100,000 women. Brazil and regions, 2001 to 2045.

Source: Brazilian Health Informatics Department ^11^ and Brazilian Institute of Geography and Statistics ^12^. Note: solid lines represent observed rates, dashed lines represent projected rates. * World standard population, 1960.

Figure 2 shows an increase in lung cancer mortality rates in women between 2001 and 2025, followed by a projected stability or slight decrease until 2045. The South Region maintains the highest rates throughout the period. The other regions present more homogeneous patterns, with less pronounced differences.

## Discussion

Projections for lung cancer mortality in Brazil for the period from 2026 to 2045 indicate a complex scenario, influenced by demographic transformations, historical patterns of smoking exposure, regional inequalities, and improvements in the quality of information on causes of death ^22^. The reduced smoking prevalence, observed in all regions of the country between 2008 and 2019, especially among men, constitutes a determining factor for the projected lung cancer mortality. However, this reduction occurred heterogeneously: while the South and Southeast regions maintain historically higher prevalences, the North and Northeast regions showed more expressive proportional drops ^23^ ^,^ ^24^ ^,^ ^25^ ^,^ ^26^. 

Information from the 2013 and 2019 Brazilian National Health Survey (PNS, acronym in Portuguese) reinforces this trend. Among women, the smoking prevalence fell from 7.7% to 6.1% in the North, from 9.9% to 7.7% in the Northeast, and from 11.5% to 10.4% in the Southeast. In the same period, reductions were also observed in the South (from 13.2% to 12.5%) and Central-West (from 11% to 9.7%). Among men, the drop was even more pronounced: in the North, it decreased from 19.3% to 15.2%, in the Northeast from 19.1% to 14.2%, and in the Southeast from 18.8% to 16.6%. The South Region also saw a significant decline, from 19% to 17% ^24^ ^,^ ^25^. These differences by region and sex are consistent with the projected mortality pattern, as the more accelerated reduction in smoking among men, especially in the North and Northeast, tends to be reflected in more intense drops in future rates in these regions. On the other hand, the maintenance of higher prevalences in the South and Southeast contributes to the persistence of high levels of projected mortality.

These regional disparities largely reflect the course of the tobacco epidemic in the country, initially characterized by increased consumption among men and, subsequently, among women. This temporal sequence is consistent with the smoking epidemics model described in the international literature, according to which tobacco consumption initially spreads among men, reaching its peak before women, who tend to present later maximum prevalences and, in general, of lower magnitude ^27^. This behavior can be attributed, in part, to increased knowledge about the harms of tobacco consumption and the progressive implementation of more structured control public policies, which differentially affect population groups.

One of the original contributions of this study consists in updating projections for lung cancer mortality by 2045, incorporating the most recent demographic data from the 2022 *Demographic Census*, in addition to disaggregated analysis by regions of the country, which provides the identification of distinct patterns and regional inequalities with higher accuracy. The study of Souza et al. ^8^ projected a sustained drop in standardized lung cancer mortality rates in men until 2040, while, among women, stability or decline was only expected to occur later, especially in younger age groups, from 2030 onwards.

Our findings confirm the downward trend in age-adjusted rates among men in all regions, notably in the South Region, which maintains the highest values throughout the period, while the North and Northeast regions show the lowest indices, with a more moderate reduction. Among women, an increase in rates is observed until 2025, followed by stability or a slight decrease until 2045, with the South Region again presenting the highest rates. This behavior suggests that the stability in lung cancer mortality rates among women in Brazil may be occurring earlier than in the projections of Souza et al. ^8^. This earlier occurrence may reflect advances in smoking reduction among younger women and possible improvements in access to early diagnosis in certain regions, signaling a change in the disease’s trajectory that should be closely followed by public health policies.

Thus, future lung cancer mortality trends should continue to reflect historical exposure patterns, albeit modulated by factors such as population aging, in addition to the boost in smoking control initiatives. Additionally, the regional heterogeneity observed in the estimates reinforces the need to consider prevention and control strategies that are more sensitive to local specificities and the continuity of monitoring for the quality of mortality data, especially in regions historically marked by underreporting or misclassification of basic causes of death.

Although age group-specific mortality rates are declining, especially among men, the absolute number of deaths continues to grow. The observation of divergent trends between crude and age-adjusted rates in the analysis of lung cancer mortality in Brazil reflects a demographic phenomenon known in cancer epidemiology. The progressive increase in crude rates may result from population aging, which leads to a higher absolute number of deaths in older age groups, in which the risk of the disease is naturally higher. However, age-adjusted rates - which eliminate the effect of changes in the population’s age structure over time - show a downward trend, indicating a possible reduction in the risk of death from lung cancer in each age group. This discrepancy is expected and should be carefully interpreted so an increase in the number of cases is not confused with an increase in risk. 

It should be noted that the age group-based stratification presented in Tables 2 and 4 provides an additional perspective similarly to an “adjustment by age”, which enables observing the behavior of specific rates within each age group over the periods. The total crude rates (Brazil and regions) increase because, regardless of birth cohort, mortality rates naturally grow with age. Moreover, the proportion of the population aged 60 years or older increases expressively over the projected period: between 2026-2030 and 2041-2045, this age group increases from 16% to 23% among men and from 19% to 27% among women (estimates based on Tabnet; https://datasus.saude.gov.br/informacoes-de-saude-tabnet/). This means that the population underlying Tables 2 and 4 is not the same over the periods, unlike age-adjusted rates, which use a fixed standard population to enable unambiguous comparisons. This finding reinforces the importance of considering standardized indicators to assess the effectiveness of public policies for control of smoking and other risk factors, in addition to showing the need for health care planning that takes into account the growing impact of population aging on the burden of cancer in the country. As noted by Mathers et al. ^21^, age-based standardization is essential for more reliable temporal and inter-regional comparisons, especially in contexts of demographic transformation.

This projected scenario for lung cancer is directly consistent with the national trend of epidemiological transition, in which cancer has been progressively surpassing cardiovascular diseases as the leading cause of death in Brazil. This change is especially evident in higher-income cities and the more developed regions of the country, such as the South and Southeast, where there is higher access to diagnosis and treatment ^28^.

Although Brazil has a Unified National Health System (SUS, acronym in Portuguese), significant inequalities persist in the access to and quality of services provided, especially in smaller municipalities with socioeconomic difficulties. These limitations reinforce the need for specific regional policies that address the social determinants of health and promote equitable access to quality prevention and care, especially in reducing premature cancer deaths. The transition in mortality profile, with cancer coming to have a predominant role, is already evident in high-income countries; however, in low- and middle-income countries, such as Brazil, this process occurs more slowly and is marked by these regional disparities ^28^.

From an etiological standpoint, smoking continues to be the main determinant of lung cancer in Brazil. Despite the significant drop in the prevalence of smokers since the 1990s, as a result of successful public policies, such as the Brazilian National Smoking Control Program ^29^, mortality still reflects past exposures, with differences in magnitude between sexes and regions of the country.

Additionally, the methodological study of Jardim et al. ^30^ on cancer incidence estimation in Brazil reinforces the importance of the quality and coverage of information systems, such as Population-Based Cancer Registries (RCBP, acronym in Portuguese) and the SIM. Regional heterogeneity in the incidence/mortality (I/M) ratio shows inequalities in diagnosis and access to treatment, with potential implications for future mortality estimates. Regions with a lower I/M ratio, such as the North and Northeast, for example, tend to present higher lethality and underdiagnosis, which can distort the real magnitude of the disease burden.

In parallel with the decline in traditional smoking, there is the rise of a new form of nicotine consumption: electronic cigarettes or vapes. These devices, often mistakenly seen as a less harmful option than conventional cigarettes, have been spreading rapidly, especially among young people ^31^. The expansion of vape use may have direct repercussions on future lung cancer rates, in addition to causing broader health impacts over time.

In Brazil, although the Brazilian Health Regulatory Agency (Anvisa, acronym in Portuguese) has prohibited the sale of electronic cigarettes since 2009 ^32^ ^,^ ^33^, the *Brazilian National Survey of School Health* (PeNSE, acronym in Portuguese) study shows that a portion of students have already tried these products: 19.1% of boys and 14.6% of girls. This situation is concerning and poses a new challenge for public policies on smoking control, as the long-term impacts of vape use - alone or in combination with traditional cigarettes - are still not fully known ^31^. This is compounded by the fact that conventional cigarettes are still sold in Brazil at one of the lowest prices in the Americas, which can facilitate the transition for young people who start using nicotine through vapes to regular cigarette consumption ^34^. 

It is fundamental that cancer control policies advance on different fronts, such as smoking prevention strategies, expanded access to early diagnosis and timely treatment, and the continuous improvement of epidemiological surveillance systems. However, some relevant challenges still persist and need to be addressed, such as the lobby for releasing the sale of electronic smoking devices and the debate on tax reform for tobacco products ^35^. In this context, the incorporation of population-based predictive models, which consider demographic, behavioral, and structural factors, represents a strategic tool for health care planning, especially by supporting public policies for reducing the burden of lung cancer in Brazil.

It is important to interpret the projections with caution, as methodological changes and improvements in data quality over time can influence the presented results. The database used for mortality information, although recognized for its quality, covers the COVID-19 pandemic period. Thus, any recent changes in the mortality profile may not be fully reflected in current projections.

In addition to the limitations inherent in mortality data, there are also specific restrictions on the use of the *Nordpred* model. The method is based on the assumption that age, period, and cohort effects have remained relatively stable over time, which does not always reflect the real dynamics of the disease, especially in contexts of changing patterns of tobacco exposure and population aging. Furthermore, longer projections, such as those for a 25-year period, are subject to greater uncertainty, as small variations in model parameters can result in substantial differences in future estimates. Another important point is that *Nordpred* does not provide confidence intervals for the projections, which limits the formal assessment of the variability associated with the estimates.

We also recognize that variability between different predictive models can lead to distinct estimates. Although we used previous information on observed rates to select the APC model with the best predictive performance, other methodological approaches could produce divergent results, which constitutes a common limitation in time-series analyses ^36^ ^,^ ^37^.

Despite this limitation, the projections provide a comprehensive and consistent view of lung cancer mortality patterns in Brazil, representing a valuable tool for health care planning and for assessing the disease’s impact on the country.

## Data Availability

The research data are available upon request to the corresponding author.
