# Supplementary material for: Projections for lung cancer mortality in Brazil by 2045: a regional analysis
Source: Cad Saude Publica. 2026 Jul 24;42:e00199725. doi: 10.1590/0102-311XEN199725 (PMC13411256; doi:10.1590/0102-311XEN199725)
Supplement: Translation

# Translation: Projeções da mortalidade por câncer de pulmão no Brasil até 2045: uma análise regional

**DOI:** 10.1590/0102-311XPT199725

**Authors:** Rejane de Souza Reis, Fernanda Cristina da Silva de Lima, Darlan Henrique Nascimento da Silva, Alfredo José Monteiro Scaff, André Salem Szklo

**Affiliations:** 1 Fundação do Câncer, Rio de Janeiro, Brasil.; 2 Instituto Nacional de Câncer, Rio de Janeiro, Brasil.

**Keywords:** Neoplasias Pulmonares, Tabagismo, Estimativas Populacionais, Mortalidade

## Abstract

O objetivo foi estimar as tendências temporais da mortalidade por câncer de pulmão no Brasil e regiões, estratificadas por sexo e faixa etária, no período de 2021 a 2045. Estudo de projeção de mortalidade com base nos óbitos por câncer de pulmão (CID-10: C33-C34) registrados no Sistema de Informações sobre Mortalidade entre 2001 e 2020, com redistribuição das causas mal definidas. Foram calculadas taxas brutas e ajustadas por idade, segundo a população padrão mundial de 1960. As projeções foram realizadas pelo modelo *Nordpred* no programa R, em quinquênios de 2021 a 2045. Houve aumento projetado no número absoluto de óbitos em ambos os sexos, especialmente nas regiões Sul e Sudeste. As taxas ajustadas por idade indicam tendência de queda entre homens em todas as regiões, apesar de permanecerem mais elevadas no Sul. Entre as mulheres, observa-se crescimento até 2025, seguido de uma estabilidade ou discreta redução até 2045, com as maiores taxas na Região Sul. O envelhecimento populacional contribui para o aumento das taxas brutas, mesmo com o declínio das taxas ajustadas. As projeções sugerem persistência das desigualdades regionais, que podem estar associadas a padrões históricos de tabagismo e diferenças no acesso ao diagnóstico e tratamento do câncer de pulmão. Os achados reforçam a necessidade de políticas regionais de prevenção e controle, incluindo o enfrentamento do tabagismo e a atenção às novas formas de consumo de nicotina, como os cigarros eletrônicos.

## Introdução

O câncer representa uma das principais causas de mortalidade em todo o mundo, sendo responsável por aproximadamente uma em cada seis mortes globais (16,8%) ^1^. Dentre os diversos tipos de câncer, o de pulmão destaca-se como a principal causa de óbito por câncer, com 1,8 milhão de mortes registradas em 2022. Estima-se que, no mesmo ano, tenham ocorrido cerca de 2,5 milhões de casos novos da doença em nível mundial ^2^.

A incidência e a mortalidade por câncer em geral vêm aumentando globalmente, impulsionadas, sobretudo, pelo envelhecimento populacional ^3^ e pela exposição a fatores de risco modificáveis, como o uso de tabaco e álcool, alimentação inadequada, sedentarismo e poluição atmosférica ^4^ ^,^ ^5^.

Além de seu impacto direto na saúde, o câncer constitui um importante desafio social e econômico do século XXI, representando uma barreira significativa para o aumento da expectativa de vida. Seus custos sociais e macroeconômicos são expressivos e variam conforme o tipo de câncer, o contexto geográfico e as disparidades de gênero ^6^.

A realidade brasileira em relação ao câncer de pulmão é preocupante, com taxas elevadas tanto de incidência quanto de mortalidade. Segundo estimativas do Instituto Nacional de Câncer (INCA), mais de 32 mil casos novos da doença foram esperados para o ano de 2025 no país ^7^. Entretanto, é importante mencionar que o Brasil é um país de dimensões continentais, marcado por grande diversidade socioeconômica, cultural e ambiental entre as suas regiões. Essas diferenças podem refletir distintos perfis de exposição aos fatores de risco, no acesso aos serviços de saúde e nas estratégias locais de prevenção e controle do câncer. Sendo assim, uma análise regional torna-se fundamental para compreender melhor o cenário epidemiológico do câncer de pulmão no país, permitindo identificar desigualdades e orientar políticas públicas mais eficazes e equitativas.

Embora já exista um estudo de projeção da mortalidade por câncer de pulmão no Brasil ^8^, ainda há uma lacuna importante na literatura, faltando análises mais recentes e detalhadas que considerem as diferenças regionais do país. Tal fato reforça a relevância e a originalidade do presente estudo.

As estimativas de casos novos e de mortalidade por câncer desempenham um papel essencial no embasamento de políticas públicas e na distribuição eficiente de recursos voltados ao enfrentamento da doença. A vigilância do câncer se mostra fundamental para o planejamento, acompanhamento e avaliação das estratégias de controle ^7^. Nesse sentido, os resultados deste estudo poderão servir de subsídios para essas políticas específicas, como, por exemplo, o Plano Nacional de Controle do Câncer, auxiliando na identificação de tendências regionais e grupos populacionais mais vulneráveis, possibilitando a melhor orientação de alocação de recursos, definição de metas, além da priorização de ações voltadas à prevenção, detecção precoce e tratamento oportuno ^9^.

No Brasil, a última década foi marcada por avanços na qualidade e na disponibilidade de informações sobre incidência e mortalidade por câncer. Inserida no contexto das ações para o enfrentamento das doenças crônicas não transmissíveis, a vigilância em câncer - sustentada por informações de registros de câncer populacionais e hospitalares, além dos dados do Sistema de Informações sobre Mortalidade (SIM) - oferece suporte para que gestores públicos possam organizar e monitorar as intervenções, além de orientar a produção científica na área ^10^.

O objetivo deste estudo foi estimar as tendências temporais previstas nas taxas de mortalidade por câncer de pulmão, considerando o país como um todo e as suas diferentes regiões geográficas, estratificadas por sexo e faixa etária. Foi utilizado, como período-base, os anos de 2001 a 2020, a partir dos quais foram analisadas as tendências da mortalidade por câncer de pulmão no Brasil. A partir dessas tendências, foram realizadas projeções para o período de 2021 a 2045, permitindo uma análise abrangente da evolução esperada da doença nas próximas décadas.

## Materiais e métodos

Trata-se de um estudo ecológico de série temporal, tendo como objetivo analisar as tendências e projeções da mortalidade por câncer de pulmão no Brasil.

As informações de mortalidade foram obtidas por intermédio das bases de dados do SIM ^11^. As populações para Brasil e regiões foram obtidas usando-se as bases de dados do Instituto Brasileiro de Geografia e Estatística (IBGE) ^12^. 

A mortalidade foi analisada de acordo com a 10ª revisão da Classificação Internacional de Doenças (CID-10), para pulmão (C33-C34) e para as mal definidas (R00-R99) ^13^. 

Este estudo apresenta projeções de mortalidade por câncer de pulmão no Brasil e nas suas regiões, desagregadas por sexo e faixa etária (0-39, 40-49, 50-59, 60-69, 70-79 anos e de 80 ou mais anos) para os anos de 2021 a 2045 agrupadas de 5 em 5 anos ^14^. As projeções foram baseadas em séries históricas de mortalidade observadas entre 2001 e 2020, que constituem o período-base do modelo. Foram estimados tanto o número absoluto de óbitos quanto as taxas de mortalidade - brutas e ajustadas por idade pela população padrão mundial de 1960, oferecendo uma visão abrangente da carga da doença no país ^14^ ^,^ ^15^. 

O denominador para calcular as taxas de mortalidade do Brasil e regiões foram as populações censitárias (2010), e para os demais anos as populações foram da projeção populacional do IBGE ^12^. 

A utilização da população padrão de Segi ^14^, segue a convenção adotada em diversos estudos epidemiológicos nacionais e internacionais, favorecendo a comparabilidade temporal com séries históricas e entre países que usam a mesma padronização ^16^ ^,^ ^17^. Embora alternativas mais recentes, como a população padrão da Organização Mundial da Saúde (OMS) 2000-2025, possam refletir melhor a estrutura etária contemporânea, sua adoção poderia dificultar comparações com estudos prévios, especialmente aqueles que analisam longas séries históricas de mortalidade. As taxas brutas refletem diretamente o número absoluto de casos em relação à população total, sendo influenciadas pelo envelhecimento populacional. Já as taxas ajustadas por idade controlam o efeito da estrutura etária, permitindo comparações mais adequadas ao longo do tempo e entre regiões. 

O modelo exige, pelo menos, 15 anos consecutivos de informações (períodos de cinco anos). As projeções foram calculadas para cada período (2021-2025; 2026-2030; 2031-2035; 2036-2040; 2041-2045) usando-se o programa *Nordpred*, dentro do programa estatístico R (http://www.r-project.org) ^18^. 

Essa função foi desenvolvida pelo Registro de Câncer da Noruega e baseia-se em uma versão do modelo *age-period-cohort* (APC), amplamente empregado na projeção de tendências de incidência e mortalidade por câncer. Os modelos APC foram ajustados considerando-se a função de ligação (*power*-5) e Poisson para avaliar o ajuste e possíveis efeitos de superdispersão. A comparação foi realizada com base no *deviance*, graus de liberdade e valor de p do modelo ^18^ ^,^ ^19^ ^,^ ^20^. Os resultados (Material Suplementar; https://cadernos.ensp.fiocruz.br/static//arquivo/supl-e00199725_4669.pdf) mostraram que ambos os ajustes apresentaram boa adequação, com razões *deviance*/graus de liberdade próximas, indicando ausência de superdispersão relevante. As diferenças entre as duas funções de ligação foram pequenas, e o modelo *power*-5 foi adotado para as projeções finais. Essa transformação suaviza a relação entre os efeitos e as taxas, reduzindo a influência de valores extremos e melhorando a estabilidade das projeções, especialmente em séries longas e com pequenas flutuações anuais.

O modelo pode ser representado pela equação: Rap = (Aa + Dp + Pp + Cc)^5^, em que Rap é a taxa de mortalidade no grupo etário *a* e no período *p*; *D* é o parâmetro de tendência comum (*drift*); *Aa*, o componente etário; *Pp*, o componente não linear de período; e *Cc*, o componente não linear de coorte ^19^ ^,^ ^20^.

Realizou-se a redistribuição das causas mal definidas de óbito (as categorias incluídas no Capítulo XVIII da CID-10: R00-R99) que seguiu a metodologia proposta por Mathers et al. ^21^, que assume que a distribuição das causas verdadeiras desses óbitos é a mesma que a dos óbitos relatados para causas naturais (não externas). Essa redistribuição foi realizada proporcionalmente entre as causas naturais (excluídas as causas externas - Capítulo XX: V01-Y98) segundo sexo, faixa etária de cinco anos e região geográfica. Para cada ano e Unidade da Federação, foram calculados fatores de correção aplicados às causas bem definidas, obtidos com base na razão entre o total de óbitos estimados e o total de óbitos informados no SIM ^21^ ^,^ ^22^. Foi aplicada apenas ao período observado, com o objetivo de ajustar a série histórica antes da modelagem e projeção. As projeções geradas pelo modelo *Nordpred* consideraram, portanto, os óbitos já corrigidos para sub-registro e causas mal definidas.

Conforme a *Resolução nº 510/2016,* do Conselho Nacional de Saúde, estudos com dados secundários, de acesso público, estão isentos da necessidade de análise ética.

## Resultados

Os resultados mostram que, tanto o número absoluto quanto as taxas brutas de mortalidade por câncer de pulmão, devem continuar aumentando até 2045, em ambos os sexos, impulsionados, principalmente, pelo envelhecimento populacional e pela crescente carga da doença nas idades mais avançadas. Entre os homens, observou-se aumento no número de óbitos por câncer de pulmão ao longo dos períodos analisados. Os óbitos passaram de 63.949 em 2001-2005 para 78.491 em 2016-2020 (Tabela 1). As projeções indicam que esse crescimento deve continuar, alcançando 133.700 óbitos em 2041-2045 (Tabela 2). Entre as mulheres, o padrão de crescimento também foi observado. O número de óbitos aumentou de 30.535 em 2001-2005 para 51.690 em 2016-2020, alcançando 121.114 óbitos em 2041-2045 (Tabelas 3 e 4). 

**Tabela 1:** Número de óbitos e taxas brutas de mortalidade observadas de câncer de pulmão por 100 mil homens. Brasil e regiões, 2001 a 2020.

Regiões/Faixa etária (anos)	Óbitos observados				Taxas brutas observadas			
	2001-2005	2006-2010	2011-2015	2016-2020	2001-2005	2006-2010	2011-2015	2016-2020
Norte								
0-39	83	102	110	100	0,29	0,34	0,35	0,31
40-49	193	175	220	188	5,70	4,39	4,77	3,50
50-59	493	547	591	669	23,58	20,99	18,62	17,91
60-69	829	913	1.053	1.280	67,44	62,16	58,01	56,16
70-79	761	881	1.132	1.283	122,36	118,93	125,30	116,15
80 ou mais	320	417	622	726	120,70	137,84	172,94	163,26
Total	2.679	3.035	3.728	4.246	7,47	7,71	8,81	9,49
Nordeste								
0-39	320	293	341	336	0,35	0,31	0,37	0,37
40-49	748	715	691	650	6,01	4,98	4,38	3,74
50-59	1.765	1.972	2.267	2.345	20,72	20,14	19,77	17,64
60-69	2.731	3.158	3.878	4.688	49,48	49,72	52,50	54,74
70-79	2.721	3.036	3.967	4.880	85,93	86,21	96,59	101,45
80 ou mais	1.434	1.831	2.393	3.154	90,40	103,48	124,27	144,18
Total	9.719	11.005	13.537	16.053	7,86	8,52	10,18	11,78
Centro-oeste								
0-39	76	76	97	72	0,32	0,31	0,38	0,28
40-49	269	279	249	218	7,27	6,44	5,09	3,98
50-59	648	744	898	871	27,75	25,83	25,74	21,22
60-69	1.081	1.262	1.528	1.706	78,00	76,64	75,63	67,87
70-79	952	1.186	1.527	1.760	144,09	142,86	149,73	142,43
80 ou mais	415	537	748	1.014	167,02	174,90	195,26	205,94
Total	3.441	4.084	5.047	5.641	10,75	11,75	13,59	14,24
Sudeste								
0-39	483	425	465	462	0,37	0,32	0,35	0,36
40-49	2.232	2.015	1.535	1.253	9,16	7,63	5,55	4,26
50-59	5.762	6.510	6.683	5.824	35,62	33,43	29,87	23,86
60-69	10.076	10.045	10.908	12.691	104,32	89,60	79,51	76,22
70-79	9.656	10.445	10.926	11.850	183,97	174,00	159,20	144,87
80 ou mais	3.643	4.756	5.977	7.508	201,35	208,80	213,75	221,35
Total	31.852	34.196	36.494	39.588	16,92	17,33	17,84	18,77
Sul								
0-39	179	188	183	176	0,40	0,42	0,42	0,40
40-49	1.053	1.022	734	575	12,28	10,90	7,60	5,77
50-59	3.057	3.486	3.543	3.168	53,03	50,51	44,29	35,85
60-69	5.504	5.804	6.219	6.924	161,24	143,68	125,19	114,68
70-79	4.852	5.621	6.086	6.548	274,38	273,60	252,34	220,60
80 ou mais	1.613	2.302	2.920	3.468	272,11	315,05	326,43	313,69
Total	16.258	18.423	19.685	20.859	25,13	27,30	28,11	28,56
Brasil								
0-39	1.141	1.084	1.196	1.146	0,36	0,33	0,37	0,36
40-49	4.495	4.206	3.429	2.884	8,57	7,20	5,48	4,27
50-59	11.725	13.259	13.982	12.877	33,61	31,83	28,83	23,68
60-69	20.221	21.182	23.586	27.289	95,35	85,69	78,86	75,71
70-79	18.942	21.169	23.638	26.321	165,20	160,98	154,44	143,84
80 ou mais	7.425	9.843	12.660	15.870	164,93	182,70	199,08	208,21
Total	63.949	70.743	78.491	86.387	14,39	15,12	16,12	17,12

**Tabela 2:** Número de óbitos e taxas brutas de mortalidade projetadas de câncer de pulmão por 100 mil homens. Brasil e regiões, 2021 a 2045.

Regiões/Faixa etária (anos)	Óbitos projetados					Taxas brutas projetadas				
	2021-2025	2026-2030	2031-2035	2036-2040	2041-2045	2021-2025	2026-2030	2031-2035	2036-2040	2041-2045
Norte										
0-39	100	99	97	95	90	0,32	0,32	0,33	0,34	0,34
40-49	213	223	220	229	236	3,42	3,28	3,15	3,17	3,14
50-59	672	710	786	852	861	15,61	14,10	13,36	13,17	12,94
60-69	1.382	1.492	1.624	1.790	2.033	50,12	45,50	42,27	39,47	38,12
70-79	1.440	1.759	2.018	2.266	2.534	107,02	102,20	93,8	87,31	82,31
80 ou mais	863	1.009	1.240	1.578	1.877	164,88	152,65	144,3	139,84	129,78
Total	4.670	5.292	5.985	6.810	7.631	10,03	10,99	12,11	13,54	15,04
Nordeste										
0-39	342	351	354	347	334	0,40	0,43	0,46	0,48	0,50
40-49	761	753	762	758	774	3,89	3,59	3,69	3,74	3,82
50-59	2.283	2.399	2.605	2.588	2.584	15,58	14,79	14,12	13,03	13,14
60-69	5.303	5.620	5.527	5.829	6.338	52,81	47,88	42,19	39,80	37,73
70-79	5.880	7.064	8.093	8.590	8.622	105,41	107,48	102,30	91,61	81,41
80 ou mais	3.710	4.638	5.808	7.109	8.339	150,87	155,34	159,65	160,34	152,19
Total	18.279	20.825	23.149	25.221	26.991	13,21	14,91	16,49	17,95	19,29
Centro-oeste										
0-39	68	50	47	47	44	0,26	0,20	0,19	0,19	0,18
40-49	213	229	209	159	158	3,46	3,47	3,12	2,34	2,29
50-59	771	709	750	843	798	16,62	13,56	12,68	13,24	12,30
60-69	1.842	1.915	1.838	1.838	2.030	60,63	53,10	44,41	39,03	37,80
70-79	1.996	2.302	2.648	2.891	2.912	131,93	120,56	111,63	101,15	87,59
80 ou mais	1.135	1.372	1.699	2.075	2.498	191,28	183,07	175,08	164,74	155,12
Total	6.025	6.577	7.191	7.853	8.440	14,44	15,08	15,88	16,84	17,70
Sudeste										
0-39	478	483	459	435	405	0,39	0,41	0,41	0,41	0,41
40-49	1.290	1.469	1.543	1.631	1.594	4,03	4,42	4,78	5,18	5,14
50-59	5.071	4.673	4.913	5.605	5.880	19,81	17,05	16,33	17,84	19,16
60-69	13.281	12.450	11.495	11.191	12.204	69,50	59,08	51,13	45,95	45,18
70-79	13.308	15.766	17.177	16.831	16.198	132,91	127,91	118,02	103,07	91,49
80 ou mais	7.811	8.900	10.773	13.345	15.210	199,21	181,32	170,45	166,21	154,92
Total	41.239	43.741	46.360	49.038	51.491	19,26	20,25	21,35	22,59	23,86
Sul										
0-39	160	156	145	137	126	0,37	0,37	0,35	0,35	0,33
40-49	633	669	659	697	677	5,86	5,78	5,59	5,87	5,77
50-59	2.518	2.263	2.543	2.801	2.862	27,48	23,76	24,47	25,10	25,12
60-69	7.149	6.743	5.862	5.716	6.557	101,75	86,05	71,19	66,16	68,96
70-79	7.186	8.217	9.030	8.946	8.151	194,34	179,66	165,27	144,68	123,83
80 ou mais	3.804	4.422	5.314	6.481	7.509	284,61	253,52	231,35	220,03	205,66
Total	21.450	22.470	23.553	24.778	25.882	28,37	28,89	29,67	30,82	32,00
Brasil										
0-39	1.148	1.139	1.102	1.061	999	0,37	0,38	0,39	0,39	0,39
40-49	3.110	3.343	3.393	3.474	3.439	4,16	4,22	4,33	4,47	4,44
50-59	11.315	10.754	11.597	12.689	12.985	19,39	16,96	16,4	16,86	17,34
60-69	28.957	28.220	26.346	26.364	29.162	68,99	59,37	50,87	46,35	45,55
70-79	29.810	35.108	38.966	39.524	38.417	134,6	129,54	120,07	105,84	93,06
80 ou mais	17.323	20.341	24.834	30.588	35.433	196,11	184,10	176,32	171,88	161,02
Total	91.663	98.905	106.238	113.700	120.435	17,75	18,83	19,98	21,26	22,51

**Tabela 3:** Número de óbitos e taxas brutas de mortalidade observadas de câncer de pulmão por 100 mil mulheres. Brasil e regiões, 2001 a 2020.

Regiões/Faixa etária (anos)	Óbitos observados				Taxas brutas observadas			
	2001-2005	2006-2010	2011-2015	2016-2020	2001-2005	2006-2010	2011-2015	2016-2020
Norte								
0-39	82	84	84	87	0,29	0,28	0,27	0,28
40-49	164	167	157	171	5,06	4,29	3,45	3,19
50-59	267	305	426	500	13,76	12,25	13,73	13,49
60-69	385	436	580	820	33,37	31,22	32,85	36,00
70-79	337	421	606	813	54,44	55,48	64,60	70,29
80 ou mais	197	262	371	499	60,02	70,53	83,43	90,53
Total	1.432	1.675	2.224	2.890	4,06	4,32	5,33	6,54
Nordeste								
0-39	258	235	274	286	0,28	0,25	0,29	0,32
40-49	554	661	755	783	4,07	4,19	4,35	4,13
50-59	1.130	1.457	2.018	2.309	11,92	13,21	15,57	15,30
60-69	1.518	2.024	2.938	3.911	23,80	27,70	34,14	38,61
70-79	1.335	1.840	2.709	3.702	34,62	41,44	52,13	61,17
80 ou mais	849	1.200	1.833	2.730	40,89	49,22	65,07	82,22
Total	5.644	7.417	10.527	13.721	4,37	5,48	7,52	9,54
Centro-oeste								
0-39	61	68	81	65	0,26	0,27	0,32	0,26
40-49	149	216	254	204	3,92	4,77	4,96	3,54
50-59	347	458	634	759	14,99	15,39	17,10	17,20
60-69	493	648	890	1.142	36,26	38,60	41,40	41,18
70-79	463	639	861	1.175	66,23	71,52	76,56	83,04
80 ou mais	258	358	560	709	83,80	93,53	114,84	110,89
Total	1.771	2.387	3.280	4.054	8,10	9,24	11,11	12,12
Sudeste								
0-39	374	418	406	386	0,28	0,31	0,31	0,30
40-49	1.522	1.709	1.461	1.272	5,79	5,94	4,86	4,01
50-59	2.852	4.004	5.264	5.151	16,14	18,51	20,87	18,64
60-69	3.857	4.980	6.557	9.078	34,14	37,88	40,52	45,65
70-79	3.939	4.984	6.043	7.487	54,69	60,93	65,40	68,71
80 ou mais	2.356	3.338	4.403	5.807	75,49	83,04	88,90	98,20
Total	14.900	19.433	24.134	29.181	7,50	9,30	11,13	13,04
Sul								
0-39	138	157	161	136	0,31	0,36	0,37	0,31
40-49	618	729	763	598	6,88	7,34	7,46	5,71
50-59	1.343	1.769	2.315	2.640	22,05	23,90	26,61	27,26
60-69	1.929	2.598	3.229	4.204	50,39	57,26	57,45	60,90
70-79	1.849	2.398	3.205	3.901	78,74	88,53	102,53	103,02
80 ou mais	911	1.298	1.852	2.432	92,16	103,22	118,98	128,99
Total	6.788	8.949	11.525	13.911	10,17	12,80	15,83	18,29
Brasil								
0-39	913	962	1.006	960	0,28	0,29	0,31	0,30
40-49	3.007	3.482	3.390	3.028	5,38	5,54	5,04	4,19
50-59	5.939	7.993	10.657	11.359	15,84	17,56	19,85	18,77
60-69	8.182	10.686	14.194	19.155	34,07	38,07	41,35	45,64
70-79	7.923	10.282	13.424	17.078	53,81	60,55	68,40	73,28
80 ou mais	4.571	6.456	9.019	12.177	67,00	76,23	87,92	98,92
Total	30.535	39.861	51.690	63.757	6,61	8,16	10,15	12,07

**Tabela 4:** Número de óbitos e taxas brutas de mortalidade projetadas de câncer de pulmão por 100 mil mulheres. Brasil e regiões, 2021 a 2045.

Regiões/Faixa etária (anos)	Óbitos projetados				Taxas brutas projetadas			
	2021-2025	2026-2030	2031-2035	2036-2040	2041-2045	2021-2025	2026-2030	2031-2035	2036-2040	2041-2045
Norte										
0-39	87	78	83	83	81	0,28	0,26	0,29	0,31	0,32
40-49	197	226	212	184	193	3,15	3,29	3,00	2,53	2,59
50-59	534	545	621	687	645	12,37	10,70	10,36	10,41	9,49
60-69	1.045	1.247	1.328	1.372	1.560	37,00	36,65	33,11	28,8	27,69
70-79	1.087	1.447	1.859	2.191	2.352	74,55	76,34	77,36	74,84	67,52
80 ou mais	675	928	1.261	1.683	2.215	102,02	111,39	115,02	115,09	116,00
Total	3.625	4.471	5.364	6.200	7.046	7,86	9,37	10,91	12,36	13,87
Nordeste										
0-39	250	216	228	224	219	0,29	0,27	0,30	0,32	0,34
40-49	861	891	730	574	582	4,05	3,91	3,25	2,64	2,75
50-59	2.490	2.584	2.716	2.670	2.197	14,97	14,17	13,21	12,08	10,06
60-69	4.889	5.666	5.835	5.871	6.138	40,90	40,39	37,42	34,13	31,46
70-79	4.988	6.395	7.826	8.769	9.005	69,73	75,17	76,51	72,27	66,20
80 ou mais	3.637	4.947	6.559	8.326	10.293	95,63	108,25	116,99	121,25	121,16
Total	17.115	20.699	23.894	26.434	28.434	11,68	13,96	15,99	17,63	19,02
Centro-oeste										
0-39	43	31	28	28	26	0,17	0,12	0,11	0,12	0,11
40-49	215	196	153	110	109	3,31	2,84	2,22	1,59	1,58
50-59	760	743	775	752	629	15,19	13,15	12,14	11,07	9,25
60-69	1.399	1.693	1.764	1.818	1.949	40,57	41,02	37,39	33,93	32,04
70-79	1.470	1.859	2.391	2.939	3.132	80,78	78,58	79,90	81,33	75,22
80 ou mais	934	1.281	1.729	2.290	3.034	115,57	120,24	120,86	120,09	122,24
Total	4.821	5.803	6.840	7.937	8.879	12,83	13,98	15,23	16,62	17,81
Sudeste										
0-39	358	330	325	310	294	0,29	0,29	0,30	0,30	0,31
40-49	1.292	1.311	1.176	1.074	1.056	3,76	3,72	3,48	3,29	3,34
50-59	4.598	4.244	4.341	4.376	3.974	15,93	13,89	13,06	12,78	12,07
60-69	11.036	11.219	10.174	9.615	9.975	47,70	44,01	37,82	33,51	31,77
70-79	9.783	13.249	16.194	16.548	15.350	72,88	79,70	82,26	75,45	65,65
80 ou mais	6.921	8.749	11.617	15.898	20.127	102,69	106,92	111,83	120,46	124,45
Total	33.988	39.102	43.827	47.821	50.776	14,91	16,96	18,87	20,56	21,93
Sul										
0-39	145	153	148	141	132	0,34	0,37	0,37	0,37	0,36
40-49	616	634	631	682	668	5,46	5,30	5,23	5,64	5,67
50-59	2.426	2.199	2.249	2.349	2.369	24,18	21,3	20,14	19,84	19,83
60-69	5.210	5.744	5.501	5.167	5.390	64,02	63,01	57,82	52,47	50,39
70-79	4.803	6.221	7.853	8.732	8.494	101,4	105,99	111,75	109,95	101,72
80 ou mais	3.081	3.997	5.127	6.839	8.964	138,08	141,43	139,9	146,22	154,53
Total	16.281	18.948	21.509	23.910	26.017	20,62	23,28	25,83	28,3	30,59
Brasil										
0-39	883	808	812	786	752	0,29	0,28	0,29	0,3	0,31
40-49	3.181	3.258	2.902	2.624	2.608	3,99	3,89	3,53	3,25	3,30
50-59	10.808	10.315	10.702	10.834	9.814	16,67	14,77	13,84	13,28	12,22
60-69	23.579	25.569	24.602	23.843	25.012	47,63	45,52	40,51	36,2	34,11
70-79	22.131	29.171	36.123	39.179	38.333	77,41	82,73	85,32	80,7	72,35
80 ou mais	15.248	19.902	26.293	35.036	44.633	107,05	113,87	118,51	124,64	128,03
Total	75.830	89.023	101.434	112.302	121.152	13,98	16,10	18,08	19,86	21,39

Ao comparar as taxas brutas no Brasil do último quinquênio observado (2016-2020) com o último quinquênio projetado (2021-2045), verifica-se um aumento importante em ambos os sexos. Entre os homens, a taxa passa de 17,12 para 22,51 por 100 mil homens, o que representa um incremento de aproximadamente 31,5% no período. Entre as mulheres, o crescimento projetado é ainda mais expressivo: a taxa aumenta de 12,07 para 21,39 por 100 mil mulheres, correspondendo a uma elevação de cerca de 77,2% (Tabelas 1, 2, 3 e 4).

Quando se analisa regionalmente, verifica-se que as regiões Sul e Sudeste apresentaram as maiores taxas brutas em todos os períodos, atingindo 32,00 e 23,86 óbitos a cada 100 mil homens no quinquênio final, respectivamente. Já o Norte mantém as menores taxas, com 10,99 a 15,04 óbitos a cada 100 mil homens entre 2026 e 2045 (Tabela 2).

Embora a taxa bruta de mortalidade entre as mulheres seja menor em relação aos homens, também há um crescimento ao longo do tempo, no Brasil. As regiões Sul e Sudeste destacam-se novamente com as maiores taxas, chegando a 30,59 e 21,93 óbitos a cada 100 mil mulheres, respectivamente, entre 2041 e 2045. Enquanto a Região Norte apresentou as menores taxas, com valores entre 9,37 e 13,87 óbitos a cada 100 mil mulheres no período de 2026 a 2045 (Tabela 4).

Em relação às taxas específicas por faixa etária, no Brasil, observam-se padrões distintos segundo idade e sexo. Entre os homens, há declínio nas faixas etárias mais jovens: por exemplo, na faixa de 40-49 anos, a taxa bruta de mortalidade cai de 8,57 em 2001-2005 para 4,44 óbitos por 100 mil homens em 2041-2045 (Tabelas 1 e 2). Entre as mulheres, comportamento semelhante é observado em alguns grupos etários; entre 40-49 anos passou de 5,38 para 3,30 óbitos por 100 mil mulheres, na faixa de 50-59 anos, a taxa reduz de 15,84 para 12,22 óbitos por 100 mil mulheres entre 2001-2005 e 2041-2045 (Tabelas 3 e 4). Em contraste, nas faixas etárias mais avançadas (acima de 70 anos), as taxas tendem a se manter elevadas, ao mesmo tempo em que o número de óbitos nessas idades cresce de forma expressiva. Esse conjunto de resultados reforça o comportamento inverso entre a redução do risco de morte dentro de vários estratos etários e o aumento do volume total de óbitos ao longo do período analisado.

Observa-se uma tendência de queda nas taxas de mortalidade por câncer de pulmão entre homens no Brasil, ajustadas por idade, em todas as regiões entre 2001 e 2045. A Região Sul apresenta, consistentemente, as maiores taxas ao longo de todo o período, apesar da redução progressiva projetada. Já as regiões Norte e Nordeste mantêm os menores índices, com declínio mais moderado (Figura 1).

**Figura 1:** Taxas de mortalidade ajustadas * por idade, câncer de pulmão, por 100 mil homens. Brasil e regiões, entre 2001 e 2045.

Fonte: Departamento de Informática do SUS ^11^ e Instituto Brasileiro de Geografia e Estatística ^12^. Nota: linhas contínuas representam taxas observadas, linhas tracejadas representam taxas projetadas. * População padrão mundial, 1960.

A Figura 2 mostra aumento nas taxas de mortalidade por câncer de pulmão em mulheres entre 2001 e 2025, seguido de uma estabilidade ou leve queda projetada até 2045. A Região Sul mantém as maiores taxas durante todo o período. As demais regiões apresentam padrões mais homogêneos, com diferenças menos acentuadas.

**Figura 2:** Taxas de mortalidade ajustadas * por idade, câncer de pulmão, por 100 mil mulheres. Brasil e regiões, entre 2001 e 2045.

Fonte: Departamento de Informática do SUS ^11^ e Instituto Brasileiro de Geografia e Estatística ^12^. Nota: linhas contínuas representam taxas observadas, linhas tracejadas representam taxas projetadas. * População padrão mundial, 1960.

## Discussão

As projeções de mortalidade por câncer de pulmão no Brasil para o período de 2026 a 2045 apontam um cenário complexo, influenciado por transformações demográficas, padrões históricos de exposição ao tabagismo, desigualdades regionais e aprimoramentos na qualidade da informação sobre as causas de óbito ^22^. A redução na prevalência do tabagismo, observada em todas as regiões do país entre 2008 e 2019, especialmente entre os homens, constitui um fator determinante para a projeção futura da mortalidade por câncer de pulmão. No entanto, essa redução ocorreu de forma heterogênea: enquanto as regiões Sul e Sudeste mantêm prevalências historicamente mais elevadas, as regiões Norte e Nordeste apresentaram quedas mais expressivas em termos proporcionais ^23^ ^,^ ^24^ ^,^ ^25^ ^,^ ^26^. 

Informações das *Pesquisas Nacionais de Saúde* (PNS) de 2013 e 2019 reforçam essa tendência. Entre as mulheres, a prevalência de tabagismo caiu de 7,7% para 6,1% no Norte, de 9,9% para 7,7% no Nordeste e de 11,5% para 10,4% no Sudeste. No mesmo período, reduções também foram observadas nas regiões Sul (de 13,2% para 12,5%) e Centro-oeste (de 11% para 9,7%). Entre os homens, a queda foi ainda mais acentuada: no Norte, reduziu de 19,3% para 15,2%, no Nordeste de 19,1% para 14,2% e no Sudeste de 18,8% para 16,6%. A Região Sul também registrou declínio importante, passando de 19% para 17% ^24^ ^,^ ^25^. Essas diferenças regionais e por sexo são coerentes com o padrão projetado de mortalidade, uma vez que a redução mais acelerada do tabagismo entre os homens, especialmente no Norte e Nordeste, tende a se refletir em quedas mais intensas nas taxas futuras nessas regiões. Por outro lado, a manutenção de prevalências mais altas no Sul e Sudeste contribui para a persistência de níveis elevados de mortalidade projetada.

Essas disparidades regionais refletem, em grande parte, o curso da epidemia do tabaco no país, caracterizada inicialmente pelo aumento do consumo entre os homens e, posteriormente, entre as mulheres. Essa sequência temporal é coerente com o modelo das epidemias do tabagismo descrito na literatura internacional, segundo o qual o consumo do tabaco se difunde inicialmente entre os homens, atingindo seu pico antes das mulheres, que tendem a apresentar prevalências máximas mais tardias e, em geral, de menor magnitude ^27^. Esse comportamento pode ser atribuído, em parte, ao aumento do conhecimento sobre os malefícios do tabaco e à implementação progressiva de políticas públicas de controle mais estruturadas, que afetam diferencialmente os grupos populacionais.

Uma das contribuições originais deste estudo reside na atualização das projeções de mortalidade por câncer de pulmão até 2045, incorporando dados demográficos mais recentes provenientes do *Censo Demográfico* de 2022, além da análise desagregada por regiões do país, o que permite identificar padrões distintos e desigualdades regionais com maior precisão. O estudo de Souza et al. ^8^ projetava queda sustentada das taxas padronizadas de mortalidade por câncer de pulmão em homens até 2040, enquanto entre as mulheres a estabilidade ou declínio só deveria ocorrer mais tardiamente, sobretudo nas faixas etárias mais jovens, a partir de 2030.

Nossos achados confirmam a tendência de queda nas taxas ajustadas por idade entre os homens em todas as regiões, destacando-se a Região Sul, que mantém os maiores valores ao longo do período, já as regiões Norte e Nordeste apresentam os menores índices, com redução mais moderada. Entre as mulheres, observa-se aumento das taxas até 2025, seguido de uma estabilidade ou discreta queda até 2045, com a Região Sul novamente apresentando as taxas mais elevadas. Esse comportamento sugere que a estabilidade nas taxas de mortalidade por câncer de pulmão entre mulheres no Brasil pode estar ocorrendo de forma mais precoce do que o projetado por Souza et al. ^8^. Essa antecipação pode refletir avanços na redução do tabagismo entre mulheres mais jovens e possíveis melhorias no acesso ao diagnóstico precoce em determinadas regiões, sinalizando uma mudança na trajetória da doença que deve ser acompanhada de perto por políticas de saúde pública.

Dessa forma, as tendências futuras de mortalidade por câncer de pulmão deverão continuar refletindo os padrões históricos de exposição, ainda que moduladas por fatores como o envelhecimento populacional, além do fortalecimento de ações de controle do tabagismo. Adicionalmente, a heterogeneidade regional observada nas estimativas reforça a necessidade de se pensar em estratégias de prevenção e controle mais sensíveis às especificidades locais, bem como da continuidade do monitoramento da qualidade dos dados de mortalidade, especialmente em regiões historicamente marcadas por sub-registro ou má classificação das causas básicas de óbito.

Embora as taxas específicas de mortalidade por faixa etária estejam em declínio, sobretudo entre os homens, o número absoluto de óbitos segue em crescimento. A observação de tendências divergentes entre as taxas brutas e as taxas ajustadas por idade na análise da mortalidade por câncer de pulmão no Brasil reflete um fenômeno demográfico conhecido na epidemiologia do câncer. O aumento progressivo das taxas brutas pode decorrer do envelhecimento populacional, o que resulta em maior número absoluto de óbitos em faixas etárias mais avançadas, nas quais o risco da doença é naturalmente mais elevado. No entanto, as taxas ajustadas por idade - que eliminam o efeito da mudança na estrutura etária da população ao longo do tempo - revelam uma tendência de queda, indicando uma possível redução do risco de morte por câncer de pulmão em cada grupo etário. Essa discrepância é esperada e deve ser cuidadosamente interpretada para que não se confunda aumento do número de casos com aumento do risco. 

Cabe destacar que a própria estratificação por faixa etária apresentada nas Tabelas 2 e 4 fornece uma perspectiva adicional semelhante a um “ajuste por idade”, permitindo observar o comportamento das taxas específicas dentro de cada grupo etário ao longo dos períodos. As taxas brutas totais (Brasil e regiões) aumentam porque, independentemente da coorte de nascimento, as taxas de mortalidade crescem naturalmente com a idade. Além disso, a proporção da população com 60 ou mais anos aumenta expressivamente ao longo do período projetado: entre 2026-2030 e 2041-2045; essa faixa etária passa de 16% para 23% entre os homens e de 19% para 27% entre as mulheres (estimativas baseadas no Tabnet; https://datasus.saude.gov.br/informacoes-de-saude-tabnet/). Isso significa que a população subjacente às Tabelas 2 e 4 não é a mesma ao longo dos períodos, diferentemente das taxas ajustadas por idade, que utilizam uma população padrão fixa para permitir comparações inequívocas. Tal achado reforça a importância de considerar indicadores padronizados para avaliar a efetividade de políticas públicas de controle do tabagismo e outros fatores de risco, além de destacar a necessidade de planejamento em saúde que leve em conta o impacto crescente do envelhecimento populacional sobre a carga do câncer no país. Como destacado por Mathers et al. ^21^, a padronização por idade é essencial para comparações temporais e inter-regionais mais fidedignas, sobretudo em contextos de transformação demográfica.

Esse cenário projetado para o câncer de pulmão dialoga diretamente com a tendência nacional de transição epidemiológica, na qual o câncer vem progressivamente superando as doenças cardiovasculares como a principal causa de morte no Brasil. Essa mudança é especialmente evidente nas cidades de maior renda e nas regiões mais desenvolvidas do país, como Sul e Sudeste, onde há maior acesso a diagnóstico e tratamento ^28^.

Embora o Brasil disponha de um Sistema Único de Saúde (SUS), persistem importantes desigualdades no acesso e na qualidade dos serviços prestados, especialmente em municípios menores e com dificuldades de recursos socioeconômicos. Essas limitações reforçam a necessidade de políticas regionais específicas que enfrentem os determinantes sociais da saúde e promovam a equidade no acesso à prevenção e ao cuidado de qualidade, sobretudo na redução das mortes prematuras por câncer. A transição do perfil de mortalidade, com o câncer assumindo papel predominante, já é evidente em países de alta renda; no entanto, em países de baixa e média renda, como o Brasil, esse processo ocorre de forma mais lenta e marcada por essas disparidades regionais ^28^.

Do ponto de vista etiológico, o tabagismo continua sendo o principal determinante do câncer de pulmão no Brasil. Apesar da expressiva queda na prevalência de fumantes desde os anos 1990, em decorrência de políticas públicas exitosas, como o Programa Nacional de Controle do Tabagismo ^29^, a mortalidade ainda reflete exposições passadas, com diferença de magnitude entre os sexos e regiões do país.

Adicionalmente, o estudo metodológico de Jardim et al. ^30^ sobre a estimativa da incidência de câncer no Brasil reforça a importância da qualidade e da cobertura dos sistemas de informação, como os Registros de Câncer de Base Populacional (RCBP) e o SIM. A heterogeneidade regional na razão incidência/mortalidade (I/M) evidencia desigualdades no diagnóstico e acesso ao tratamento, com potenciais implicações para as estimativas de mortalidade futura. Regiões com menor razão I/M, como o Norte e o Nordeste, por exemplo, tendem a apresentar maior letalidade e subdiagnóstico, podendo distorcer a real magnitude da carga da doença.

Paralelamente à queda do tabagismo tradicional, observa-se o surgimento de uma nova forma de consumo de nicotina: os cigarros eletrônicos ou *vapes*. Esses dispositivos, muitas vezes vistos, de forma equivocada, como uma opção menos nociva que os cigarros convencionais, têm se difundido rapidamente, sobretudo entre o público jovem ^31^. A expansão do uso dos *vapes* pode ter repercussões diretas sobre as taxas futuras de câncer de pulmão, além de gerar impactos mais amplos na saúde ao longo do tempo.

No Brasil, embora a Agência Nacional de Vigilância Sanitária (Anvisa) tenha proibido a venda de cigarros eletrônicos desde 2009 ^32^ ^,^ ^33^, o estudo da *Pesquisa Nacional de Saúde do Escolar* (PeNSE) revela que uma parcela de estudantes já experimentou esses produtos: 19,1% dos meninos e 14,6% das meninas. Esse cenário preocupa e impõe um novo desafio às políticas públicas de controle do tabagismo, uma vez que os impactos de longo prazo do uso dos *vapes* - isoladamente ou em combinação com o cigarro tradicional - ainda não são totalmente conhecidos ^31^. Junta-se a isso o fato de que o cigarro convencional, no Brasil, ainda é vendido a um dos preços mais baixos das Américas, o que pode facilitar a transição de jovens que começam a usar nicotina pelos *vapes* para o consumo regular de cigarros ^34^. 

É fundamental que as políticas de controle do câncer avancem em diferentes frentes, como as estratégias de prevenção do tabagismo, a ampliação do acesso ao diagnóstico precoce e ao tratamento oportuno, e a melhoria contínua dos sistemas de vigilância epidemiológica. No entanto, ainda persistem alguns desafios relevantes e que precisam ser enfrentados, como, por exemplo, a pressão pela liberação da comercialização dos dispositivos eletrônicos para fumar e o debate sobre a reforma tributária do imposto para produtos derivados do tabaco ^35^. Nesse contexto, a incorporação de modelos preditivos de base populacional, que considerem fatores demográficos, comportamentais e estruturais representa uma ferramenta estratégica para o planejamento em saúde, especialmente ao subsidiar políticas públicas voltadas à redução da carga do câncer de pulmão no Brasil.

É importante interpretar as projeções com cautela, uma vez que alterações metodológicas e melhorias na qualidade dos dados ao longo do tempo podem influenciar os resultados apresentados. A base de dados utilizada para as informações de mortalidade, embora reconhecida pela sua qualidade, abrange o período da pandemia de COVID-19. Assim, eventuais mudanças recentes no perfil da mortalidade podem não estar plenamente refletidas nas projeções atuais.

Além das limitações inerentes aos dados de mortalidade, há também restrições específicas ao uso do modelo *Nordpred*. O método baseia-se na suposição de que os efeitos de idade, período e coorte permaneceram relativamente estáveis ao longo do tempo, o que nem sempre reflete a dinâmica real da doença, especialmente em contextos de mudanças nos padrões de exposição ao tabaco e envelhecimento populacional. Ademais, projeções mais longas, como as realizadas para um período de 25 anos, estão sujeitas a maior incerteza, uma vez que pequenas variações nos parâmetros do modelo podem resultar em diferenças substanciais nas estimativas futuras. Outro ponto importante é que o *Nordpred* não fornece intervalos de confiança para as projeções, o que limita a avaliação formal da variabilidade associada às estimativas.

Também reconhecemos que a variabilidade entre diferentes modelos preditivos pode levar a estimativas distintas. Embora tenhamos utilizado informações prévias das taxas observadas para selecionar o modelo APC com melhor desempenho preditivo, outras abordagens metodológicas poderiam gerar resultados divergentes, o que constitui uma limitação comum em análises de séries temporais ^36^ ^,^ ^37^.

Apesar dessa limitação, as projeções fornecem uma visão abrangente e consistente dos padrões da mortalidade por câncer de pulmão no Brasil, representando uma ferramenta valiosa para o planejamento em saúde e para a avaliação do impacto da doença no país.

## Data Availability

Os dados de pesquisa estão disponíveis mediante solicitação à autora de correspondência.
